# The complete chloroplast genome of *Ligusticopsis acaulis* (Shan et Sheh) Pimenov (Apiaceae), an endemic species from China

**DOI:** 10.1080/23802359.2023.2191750

**Published:** 2023-03-28

**Authors:** Junmei Niu, Xinyue Wang, Jiarui Yue, Shilin Zhou, Zhenwen Liu, Jing Zhou

**Affiliations:** a School of Pharmaceutical Science and Yunnan Key Laboratory of Pharmacology for Natural Products, Kunming Medical University, Kunming, China; b Yunnan Academy of Forestry and Grassland, Kunming, China

**Keywords:** *Ligusticopsis acaulis*, complete chloroplast genome, phylogenetic analyses

## Abstract

*Ligusticopsis acaulis*, belonging to the family Apiaceae (Umbelliferae), is endemic to China. The complete chloroplast genome sequence of *L. acaulis* was assembled and annotated for the first time in this study. The results showed that the plastome was 148,509 bp in length and consisted of a pair of inverted repeat regions (IRs: 19,468 bp), a large single-copy region (LSC: 91,902 bp), and a small single-copy region (SSC: 17,671 bp). A total of 114 unique genes were annotated, including 80 protein-coding, 30 tRNA, and four rRNA genes. According to the phylogenetic analysis, *L. acaulis* belongs to the tribe Selineae, with a close relationship to *Ligusticum hispidum* (Franch.) Wolff.

## Introduction

*Ligusticopsis* was originally established by Leute in 1969, with the genus containing 14 species from China (Leute 1969). *Ligusticopsis acaulis*, a species endemic to China, has diverse chemical composition and is used as a substitute for Peucedani Radix in the Yunnan Province (Rao et al. 1995). The species was first described by Shan et al. (1986) and was named *Peucedanum acaule* Shan et Sheh. Pimenov (2017) classified *Ligusticopsis acaulis* as a synonym of *P. acaule*. The genus *Peucedanum* L. (Apiaceae) contains approximately 120 species widely distributed in Eurasia and Africa (Pimenov and Leonov 1993). There are about 40 species of *Peucedanum* in China, with 33 species being endemic (Sheh and Watson 2005). However, due to the complex species composition and ambiguous relationship with related taxa, the taxonomy of *Peucedanum* has long been contentious.

In recent years, the chloroplast (cp) genome was applied to the phylogenetic reconstruction of Apiaceae, whereby a few contentious relationships were successfully resolved. Here, the complete cp genome sequence of *L. acaulis* was characterized, and its phylogenetic relationships with related taxa were established.

## Materials and methods

### Sample collection, DNA extraction, and sequencing

**Figure 1.**
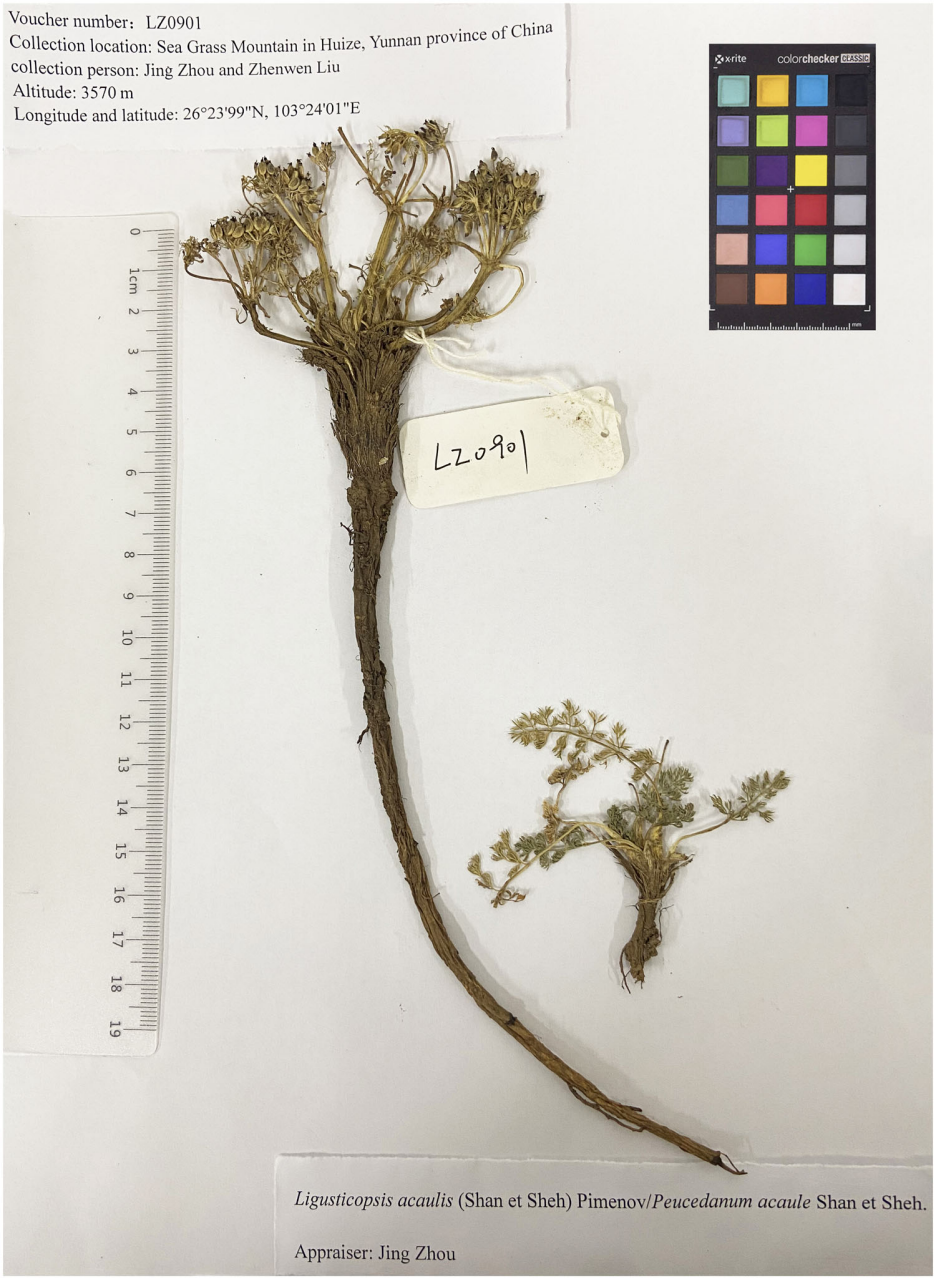
Morphological characteristics of *L. acaulis*. Herbs are perennial, 5–10 cm tall, long-cylindric root, 20–24 cm long. The specimen image was taken by the author Junmei Niu in School of Pharmaceutical Sciences and Yunnan Key Laboratory of Pharmacology for Natural Products, Kunming Medical University in September 2022, without any copyright issues.

The specimen was collected from the Sea Grass Mountain in Huize, Yunnan Province of China (26°23′99″N, 103°24′01″E, and altitude 3570 m), and the voucher was deposited in the School of Pharmaceutical Sciences and Yunnan Key Laboratory of Pharmacology for Natural Products, Kunming Medical University (appraiser: Jing Zhou; contact person: Jing Zhou, zhoujing_apiaceae@163.com) under voucher number LZ0901 (Figure 1). The modified CTAB method was used to extract total genomic DNA from leaf tissue (Doyle and Doyle 1987).

### Chloroplast genome assembly and annotation

The genome was sequenced using the Illumina HiSeq 2500 platform (Majorbio, Shanghai, China) with the paired-end (2 × 300 bp) library. The raw reads were filtered using Trimmomatic with default parameters set to remove the adapter and low-quality sequences (Bolger et al. 2014). Clean data were then assembled into circular contigs using GetOrganelle v.1.7.5.3 (Jin et al. 2020). Finally, using *Peucedanum praeruptorum* Dunn. (GenBank accession number: MN016968) as a reference, the plastome was annotated by PGA (Qu et al. 2019) and manually modified in Geneious (Kearse et al. 2012). The circular genome map was drawn by CPGView (www.1kmpg.cn/cpgview/).

### Phylogenetic analyses

A total of 31 published plastome sequences of Apiaceae from NCBI were acquired to investigate the phylogenetic position of *L. acaulis*. All sequences were aligned using MAFFT (Katoh and Standley 2013) with *Bupleurum boissieuanum* H. Wolff as the outgroup. The molecular phylogenetic tree was constructed using the maximum-likelihood (ML) analysis in RAxML (Stamatakis 2014) with 1000 rapid bootstrap replicates.

## Results and discussion

### Chloroplast genome features of *L. acaulis*

**Figure 2.**
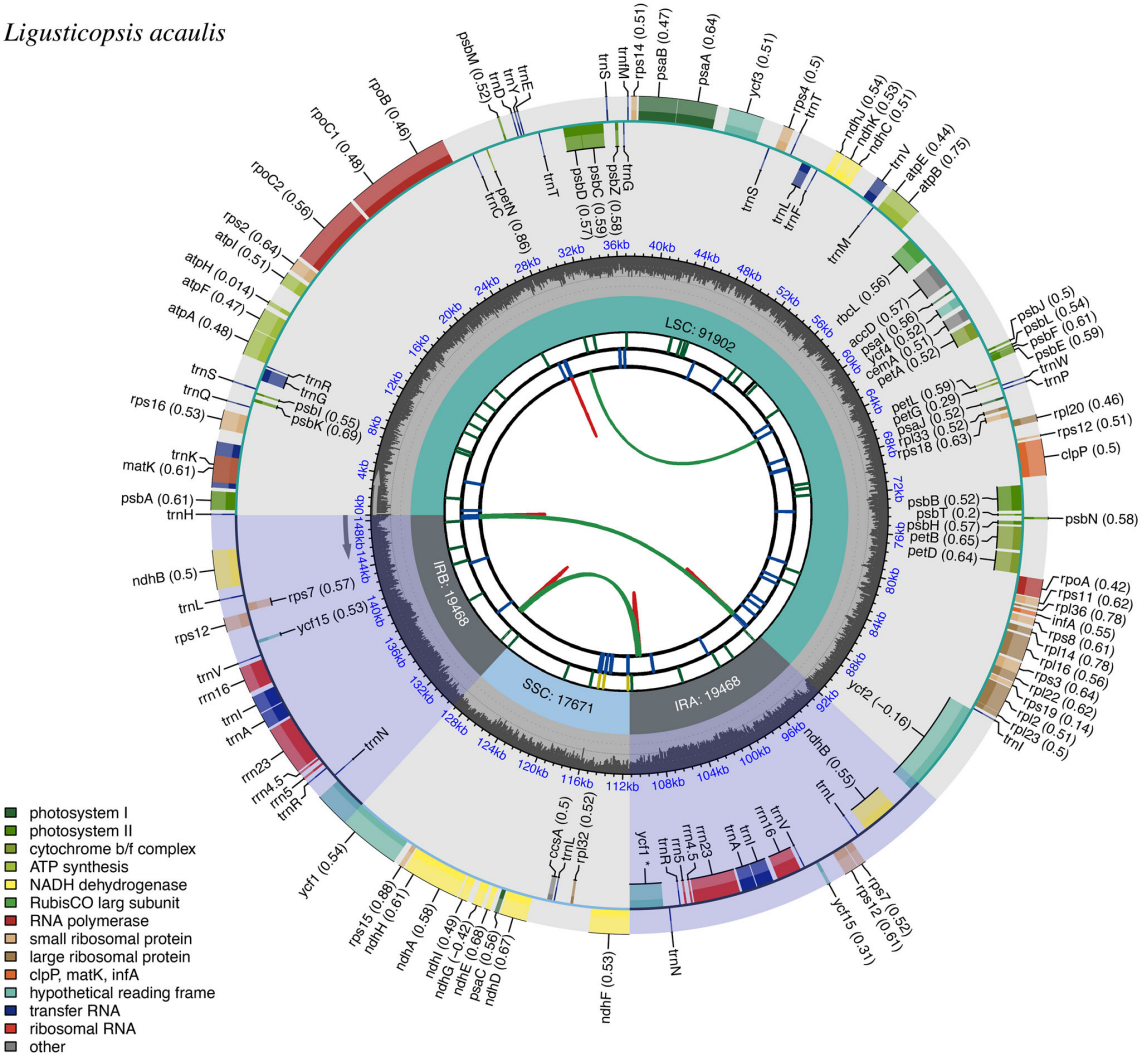
Genomic map of the *L. acaulis* chloroplast genome generated by CPGView. Genes outside the circle are transcribed in a counterclockwise direction and those inside in a clockwise direction. LSC: large single-copy; SSC: small single-copy; IR: inverted repeat. The inner circle’s dashed region represents the GC content of the chloroplast genome of *L. acaulis*. Genes belonging to different functional groups are represented using different colors.

The total length of the cp genome of *L. acaulis* was 148,509 bp and consisted of a pair of inverted repeat regions (IRs: 19,468 bp), a large single-copy region (LSC: 91,902 bp) and a small single-copy region (SSC: 17,671 bp). The overall GC content of the plastome was 37.4%, with 35.9%, 30.8%, and 43.7% in LSC, IRs, and SSC, respectively. The plastome contained 114 unique genes, including 80 protein-coding genes, 30 tRNA genes, and four rRNA genes. Of the 114 unique genes, 18 genes (including 12 protein-coding genes and six tRNA genes) had introns, with 16 genes (*atp*F, *ndh*A, *ndh*B, *pet*B, *pet*D, *rpl*2, *rpl*16, *rpo*C1, *rps*12, *rps*16, *trnA*-UGC, *trnG*-UCC, *trnI*-GAU, *trnK*-UUU, *trnL*-UAA, and *trnV*-UAC) having one intron each and two genes (*ycf*3 and *clp*P) having two introns each (Figure 2, Table 1).

**Table 1.** List of genes found in the *Ligusticopsis acaulis* chloroplast genome.

Category for genes	Group of genes	Name of genes
Photosynthesis	Rubisco	*rbcL*
	Subunit of photosystem I	*psaA*, *psaB*, *psaC*, *psaI*, *psaJ*
	Subunit of photosystem II	*psbA*, *psbB*, *psbC*, *psbD*, *psbE*, *psbF*, *psbH*, *psbI*, *psbJ*, *psbK*, *psbL*, *psbM*, *psbN*, *psbT*, *psbZ*
	Subunit of ATP synthase	*atpA*, *atpB*, *atpE*, *atpF*^a^, *atpH*, *atpI*
	Subunit of cytochrome b/f complex	*petA*, *petB*^a^, *petD*^a^, *petG*, *petL*, *petN*
	Subunit of NADPH dehydrogenase	*ndhA*^a^, *ndhB*^a^*(×2)*, *ndhC*, *ndhD*, *ndhE*, *ndhF*, *ndhG*, *ndhH*, *ndhI*, *ndhJ*, *ndhK*
Self-replication	Large subunit of ribosome	*rpl2*^a^, *rpl14*, *rpl16*^a^, *rpl20*, *rpl22*, *rpl23*, *rpl32*, *rpl33*, *rpl36*
	DNA dependent RNA polymerase	*rpoA*, *rpoB*, *rpoC1*^a^, *rpoC2*
	Small subunit of ribosome	*rps2*, *rps3*, *rps4*, *rps7(×2)*, *rps8*, *rps11*, *rps12*^a^*(×2)*, *rps14*, *rps15*, *rps16*^a^, *rps18*, *rps19*
	tRNA genes	*trnA-UGC*^a^*(×2)*, *trnC-GCA*, *trnD-GUC*, *trnE-UUC*, *trnF-GAA*, *trnfM-CAU*, *trnG-GCC*, *trnG-UCC*^a^, *trnH-GUG*, *trnI-GAU*^a^*(×2)*, *trnI-CAU(×2)*, *trnK-UUU*^a^, *trnL-CAA(×2)*, *trnL-UAA*^a^, *trnL-UAG*, *trnM-CAU*, *trnN-GUU(×2)*, *trnP-UGG*, *trnQ-UUG*, *trnR-ACG(×2)*, *trnR-UCU*, *trnS-GCU*, *trnS-GGA*, *trnS-UGA*, *trnT-GGU*, *trnT-UGU*, *trnV-GAC(×2)*, *trnV*^a^*-UAC*, *trnW-CCA*, *trnY-GUA*
	rRNA genes	*rrn5(×2)*, *rrn4.5(×2)*, *rrn16(×2)*, *rrn23(×2)*
Other genes	Subunit of acetyl-CoA-carboxylase	*accD*
	Maturase	*matK*
	Translational initiation factor	*infA*
	Protease	*clp*P^b^
	c-type cytochrome synthesis gene	*ccsA*
	Envelope membrane protein	*cemA*
Unknown function	Conserved open reading frames	*ycf1*, *ycf2*, *ycf3*^b^*, ycf4*, *ycf15*(×2)

^a^ Genes with one intron.

^b^ Genes with two introns.

### Phylogenetic analysis

**Figure 3.**
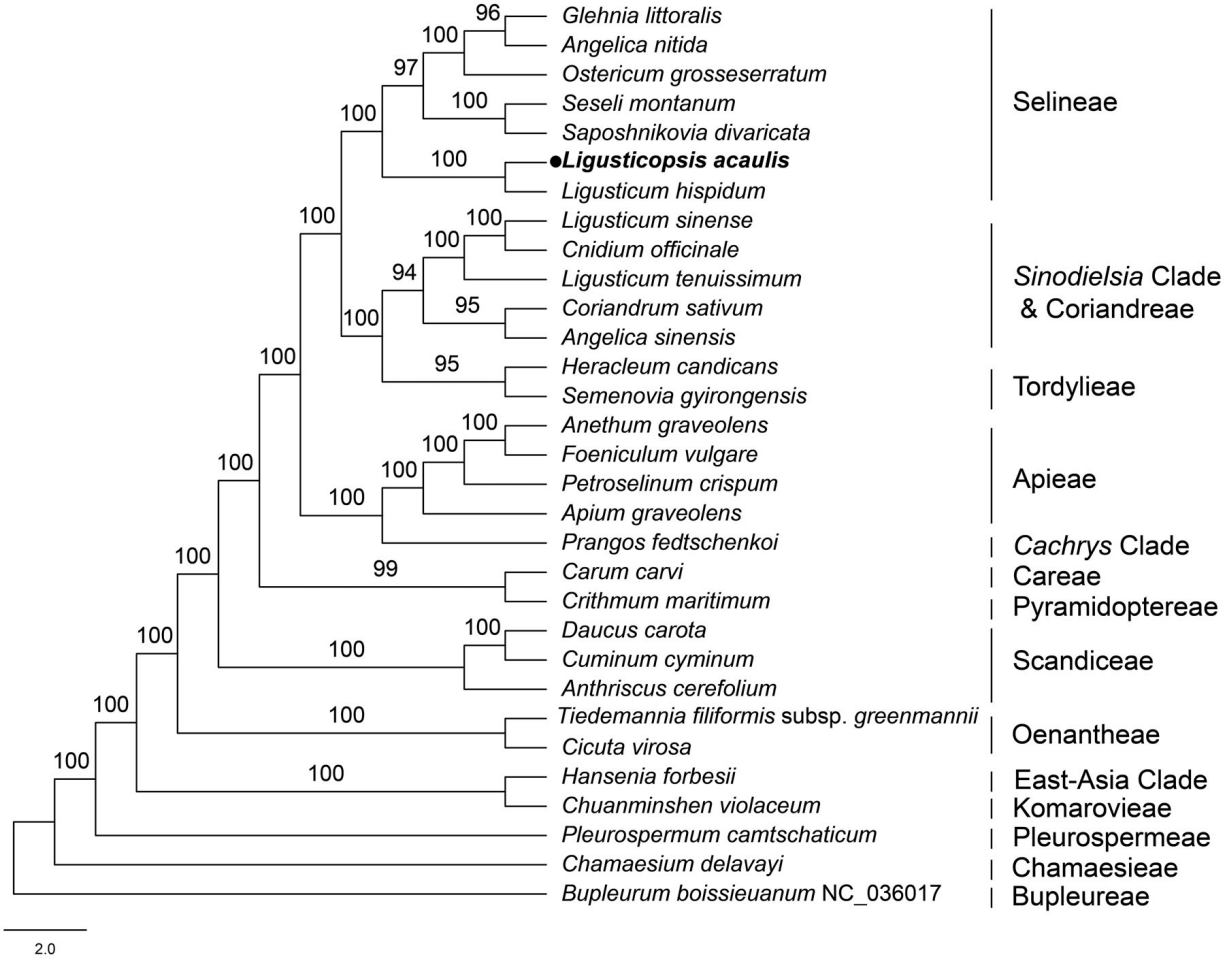
Phylogenetic tree of 31 species in the family Apiaceae based on the complete chloroplast sequences (bootstrap support values are shown above each node). The tree was constructed by ML analysis using RAxML and the GTR + G+I nucleotide model, with 1000 rapid bootstrap replicates. The following sequences were used: *G. littoralis* NC_034645 (Lee et al. 2019), *A. nitida* MF594405 (Deng et al. 2017), *O. grosseserratum* NC_028618 (Choi et al. 2016), *S. montanum* NC_027451 (Samigullin et al. 2016), *S. divaricata* MN539269 (Bao et al. 2019), *L. hispidum* MT409614 (Ren et al. 2020), *L. sinense* NK9214541 (Wu et al. 2020), *C. officinale* NC_039760 (Park et al. 2018), *L. tenuissimum* NC_029394 (www.ncbi.nlm.nih.gov/), *C. sativum* NC_029850 (www.ncbi.nlm.nih.gov/), *A. sinensis* MH430891 (Tian et al. 2019), *H. candicans* NC_045184 (Kang et al. 2019), *S. gyirongensis* NC_042912 (Xiao et al. 2019), *A. graveolens* NC_029470 (Peery 2015), *F. vulgare* NC_029469 (www.ncbi.nlm.nih.gov/), *P. crispum* NC_015821 (Downie and Jansen 2015), *A. graveolens* NC_041087 (www.ncbi.nlm.nih.gov/), *P. fedtschenkoi* KY652265 (Mustafina et al. 2019), *C. carvi* NC_029889 (www.ncbi.nlm.nih.gov/), *C. maritimum* NC_015804 (Downie and Jansen 2015), *D. carota* NC_008325 (Ruhlman et al. 2006), *C. cyminum* MN901636 (www.ncbi.nlm.nih.gov/), *A. cerefolium* NC_015113 (Downie and Jansen 2015), *T. filiformis* subsp. *greenmannii* HM596071 (Downie and Jansen 2015), *C. virosa* NC_037711 (www.ncbi.nlm.nih.gov/), *H. forbesii* NC_035054 (www.ncbi.nlm.nih.gov/), *C. violaceum* KU921430 (www.ncbi.nlm.nih.gov/), *P. camtschaticum* NC_033343 (www.ncbi.nlm.nih.gov/), *C. delavayi* MN119367 (Guo et al. 2020), and *B. boissieuanum* NC_036017 (Wu et al. 2018).

To confirm the phylogenetic position of *L. acaulis*, an ML phylogenetic tree was constructed using 31 cp genome sequences from NCBI. Using MAFFT, sequences were aligned. *B. boissieuanum* served as the outgroup. Our phylogeny showed that *L. acaulis* was closely related to *Ligusticum hispidum* and belonged to the tribe Selineae (Figure 3). Meanwhile, the phylogenetic inference provided an opportunity to delimit the species of the genus *Ligusticopsis* and understand their evolutionary relationships. The complete cp genome of *L. acaulis* provided important information for the phylogenetic analysis of the genus *Ligusticopsis* and laid the foundation for further reconstruction of the Apiaceae phylogeny.

## Supplementary Material

Supplemental Material

Supplemental Material

Supplemental Material

Supplemental Material

## Data Availability

Genome sequence data that support the findings of this study are openly available in GenBank of NCBI at https://www.ncbi.nlm.nih.gov/ under accession no. ON359911. The associated BioProject, SRA, and Bio-Sample numbers are PRJNA849278, SRR19693381, and SAMN28870233, respectively.
