# Supplementary material for: The complete chloroplast genome of Ligusticopsis acaulis (Shan et Sheh) Pimenov (Apiaceae), an endemic species from China
Source: Mitochondrial DNA B Resour. 2023 Mar 28;8(3):451–6. doi: 10.1080/23802359.2023.2191750 (PMC10062233; doi:10.1080/23802359.2023.2191750)
Supplement: Supplemental Material [file TMDN_A_2191750_SM9309.pdf]

TOPEDIT

TOPEDIT SCIENTIFIC EDITING

CERTIFICATE OF ENGLISH COPYEDITING

This is to certify that the manuscript detailed below has been edited by multiple native English-speaking academic editors in TopEdit. Neither the research content nor the authors' intentions were altered in any way during the editing process. TopEdit guarantees the quality of English grammar, spelling, punctuation, syntax, technical accuracy, and consistency in this manuscript, provided that our editors' corrections and suggestions are accepted and further changes made by the authors are checked by our editors.

Manuscript title

The complete chloroplast genome of Ligusticopsis acaulis (Shan et Sheh) Pimenov (Apiaceae), an endemic species fro m China

Date Issued

11/25/2022

Certificate Number

CN 10360-06-1125-01

TopEdit specializes in comprehensive evaluation and academic editing of technical manuscripts, book chapters, grant proposals, and other types of scientific materials. Aiming at breaking language barriers and advancing research publication, we offer four levels of English editing: Proofreading, Copyediting, Line editing, and Developmental editing. Our editorial team comprises professional native English-speaking experts in various academic fields with advanced Ph.D. degrees.

info@topeditsci.com
https://www.topeditsci.com
https://en.topeditsci.com/
