# Supplementary figures and images for: The complete chloroplast genome of Ligusticopsis acaulis (Shan et Sheh) Pimenov (Apiaceae), an endemic species from China
Source: Mitochondrial DNA B Resour. 2023 Mar 28;8(3):451–6. doi: 10.1080/23802359.2023.2191750 (PMC10062233; doi:10.1080/23802359.2023.2191750)

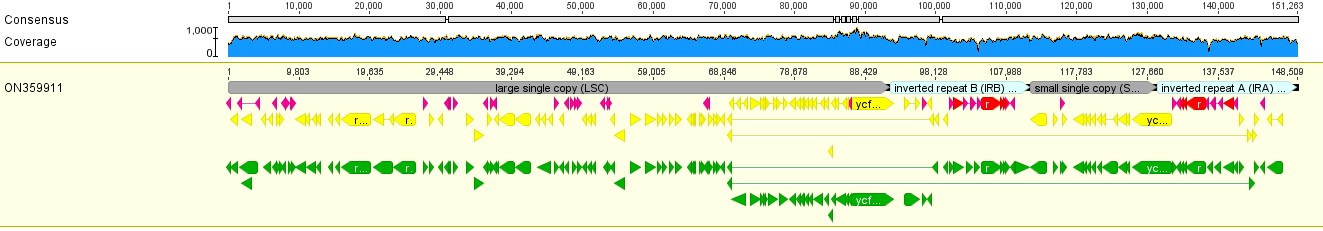

Supplement: Supplemental Material [file TMDN_A_2191750_SM9308.jpg]

| 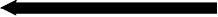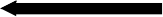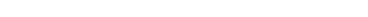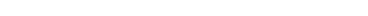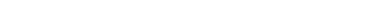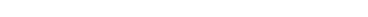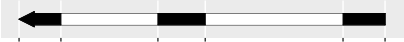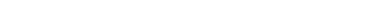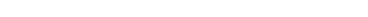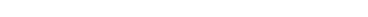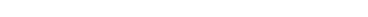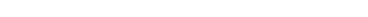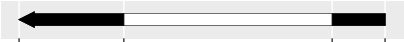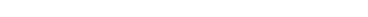  Cis−splicing Genes   | 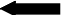 | 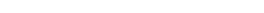 | 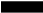 | | --- | --- | --- | |  |  |  |   ***5952*** ***5991***  ***4903***  ***5099***   | 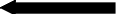 |  | 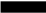 | | --- | --- | --- | |  |  |  |   ***10903***  ***11304***  ***12015*** ***12158***   |  |  | 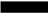 | | --- | --- | --- | |  |  |  |   ***20126***  ***21736***  ***22501*** ***22953***   | 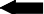 |  | 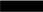 |  | 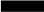 | | --- | --- | --- | --- | --- | |  |  |  |  |  |   ***42853*** ***43005*** ***43782*** ***44009*** ***44727*** ***44852***  ***70166***  ***70791***  ***71082***  ***71930*** ***72000***  ***69936***   |  |  | 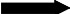 | | --- | --- | --- | |  |  |  |   Subgene  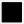 Exon 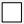 Intron  ***75716***  ***74962***  ***74967***  ***76357***   |  |  | 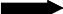 | | --- | --- | --- | |  |  |  |   ***76544***  ***77288***  ***77762***  ***76537***   | 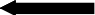 |  |  | | --- | --- | --- | |  |  |  |   ***81257***  ***81655***  ***82627*** ***82635***   | 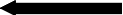 |  |  | | --- | --- | --- | |  |  |  |   ***84332***  ***84765***  ***85443*** ***85833***   |  |  | 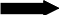 | | --- | --- | --- | |  |  |  |   ***96110***  ***94672***  ***95355***  ***93896***  ***121448***  ***121986***  ***123056*** ***123614***   |  |  |  | | --- | --- | --- | |  |  |  |   **rps16**  **atpF**  **rpoC1**  **ycf3**  **clpP**  **petB**  **petD**  **rpl16**  **rpl2**  **ndhB**  **ndhA**  **ndhB**  ***145057***  ***144302***  ***145740***  ***146516*** |
| --- | --- | --- | --- | --- | --- | --- | --- | --- | --- | --- | --- | --- | --- | --- | --- | --- | --- | --- | --- | --- | --- | --- | --- | --- | --- | --- | --- | --- | --- | --- | --- | --- | --- | --- | --- | --- | --- | --- | --- | --- | --- | --- | --- | --- | --- | --- | --- | --- | --- | --- | --- | --- | --- | --- | --- | --- | --- | --- | --- | --- | --- | --- | --- | --- |

Supplement: Supplemental Material [file TMDN_A_2191750_SM9307.docx]
