# Supplementary material for: The complete chloroplast genome of Ligusticopsis acaulis (Shan et Sheh) Pimenov (Apiaceae), an endemic species from China
Source: Mitochondrial DNA B Resour. 2023 Mar 28;8(3):451–6. doi: 10.1080/23802359.2023.2191750 (PMC10062233; doi:10.1080/23802359.2023.2191750)
Supplement: Supplemental Material [file TMDN_A_2191750_SM9306.docx]

| 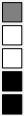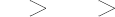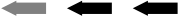  Trans−splicing Genes  69676  69789  141846  142077  142614  142639  69676  69789  97773  97798  98335  98566  141846  142077  142614  142639  69676  69789  98335  98566  97773  97798   \| 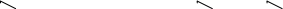 \| \| \| --- \| --- \| \| 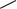 \| 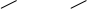 \|  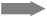 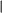 exon1 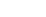 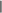 exon2 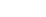 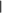 exon3  Transcript 1  Genome (+) (−)  Transcript 2  ~~exon2 exon3~~   \| 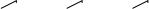 \| \| --- \| \| 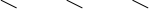 \|   exon1 exon3 exon2 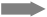 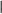 exon1 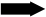 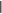 exon2 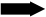 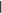 exon3  Exon  exon1 exon2 (IRa) exon3 (IRa) exon2 (IRb) exon3 (IRb)   \|  \| \| \| \| --- \| --- \| --- \| \|  \|  \|  \| |
| --- | --- | --- | --- | --- | --- | --- | --- | --- | --- | --- | --- | --- |
